# Pre-conception lifestyle intervention to optimise maternal health for a better start to life: the BEGIN BETTER prospective randomised controlled trial protocol

**DOI:** 10.1136/bmjopen-2025-105478

**Published:** 2025-10-15

**Authors:** Megan Mitchell, Andrea R Deussen, Jennie Louise, Amanda J Poprzeczny, Amy K Keir, Annette L Briley, Melissa Oxlad, Jodie M Dodd

**Affiliations:** 1 Robinson Research Institute, Department of Obstetrics and Gynaecology, The University of Adelaide, Faculty of Health and Medical Sciences, Adelaide, South Australia, Australia; 2 South Australian Health and Medical Research Institute Limited, Adelaide, South Australia, Australia; 3 Women’s and Children’s Hospital Research Centre, Adelaide, South Australia, Australia; 4 Women’s and Babies Division, Department of Obstetrics and Gynaecology, Women's and Children’s Hospital Adelaide, North Adelaide, South Australia, Australia; 5 College of Medicine and Public Health, Discipline of Paramedicine, Flinders University, Adelaide, South Australia, Australia; 6 Caring Futures Insitiute, College of Nursing and Health Sciences, Flinders University, Adelaide, South Australia, Australia; 7 School of Psychology, Faculty of Health and Medical Sciences, The University of Adelaide, Adelaide, South Australia, Australia

**Keywords:** Randomized Controlled Trial, NUTRITION & DIETETICS, Obesity, PUBLIC HEALTH, OBSTETRICS, Pregnancy

## Abstract

**Introduction:**

Overweight and obesity impacts approximately 50% of pregnant women. Professional medical colleges worldwide recommend women with a higher body mass index (BMI) lose weight before conception. While diet and lifestyle interventions before pregnancy are associated with improvements in diet and modest weight loss, subsequent clinical pregnancy outcomes are poorly reported.

Our aim is to conduct a randomised controlled trial of a comprehensive lifestyle intervention for women with overweight or obesity who are planning pregnancy. We will evaluate the impact of this intervention on maternal health and well-being prior to conception; and pregnancy, birth and newborn health outcomes in a subsequent pregnancy.

**Methods and analysis:**

Women with a BMI ≥25.0 kg/m^2^ who plan to conceive within 2 years will be recruited.

Women randomised to the ‘Educational Control Group’ will attend a pre-conception health consultation with a research midwife, providing limited information about obesity and associated risks in pregnancy, nutrition, exercise and weight management.

Women randomised to the ‘Pre-pregnancy Lifestyle Intervention Group’ will attend a pre-conception health consultation with a research midwife, as above, and additionally consult with a research dietitian and trained health coaches throughout the 6-month intervention period. Women will also have access to a specifically designed mHealth application providing tailored content and interactive tasks delivered bi-weekly during this time.

**The primary outcome is infant birth weight z-score:**

Secondary outcomes will include a range of maternal pre-conception health outcomes; maternal and infant pregnancy and birth outcomes; diet and physical activity changes; and quality of life.

We estimate a mean birth weight z-score of 0.43 (SD 1.09) and will recruit 800 women to detect 0.4 SD difference (alpha 0.05 (two-tailed); power 80%). Analyses will be intention to treat with estimates reported as relative risks and 95% CIs.

**Ethics and dissemination:**

The study protocol was approved by the Human Research and Ethics Committee of the Women’s and Children’s Hospital, Adelaide, South Australia (HREC/17/WCHN/177; 2020/HRE01445) on 17 August 2018. The first participant was recruited in June 2021, with recruitment anticipated through 2025. The study results will be disseminated in open-access international journals, scientific meetings and conferences with stakeholders.

**Trial registration number:**

ACTRN 12621000128897. This study has been registered at (https://www.anzctr.org.au/).

**STRENGTHS AND LIMITATIONS OF THIS STUDY**

- The protocol presents a proposed study design of a prospective, randomised controlled trial that will provide robust evidence on the effect of dietary and lifestyle intervention before pregnancy.
- The primary outcome is infant birth weight z-score, which is an indicator of future child obesity risk and controls for any confounding due to any effect of the intervention on gestational age at birth.
- The protocol outlines that the study will use standardised questionnaires and outcomes measurements to ensure accurate and reliable results in evaluating the clinical significance of pre-conception intervention.
- Engagement with women from inception to design, conduct and eventual dissemination increases the relevance and impact of results from the Begin Better trial.
- Participation is voluntary and recruitment is initiated through social media, and while this may limit the generalisability of the findings, this will be determined at the completion of the trial and any limitations acknowledged.

## Introduction

 Overweight and obesity are significant public health concerns, impacting approximately 50% of women entering pregnancy.^1 2^ There are well-recognised adverse pregnancy complications associated with overweight and obesity, including hypertension, pre-eclampsia,^3^ gestational diabetes mellitus (GDM),^3^ perinatal death,^4^ induction of labour^3^ and caesarean birth.^3^ Available literature^5^,^9^ consistently indicates that while providing lifestyle intervention during pregnancy can improve dietary and physical activity behaviours, there is a very modest effect on gestational weight gain (GWG), and little to no impact on clinical pregnancy and birth outcomes.^10^

Many professional colleges^211^,^15^ recommend that women with a higher body mass index (BMI) be counselled before conception and encouraged to ‘lose weight’. For women who are planning pregnancy, a significant proportion initiate changes in health behaviours, including diet, before conception.^16^ We have conducted two systematic reviews assessing weight loss interventions prior to^17^ and between pregnancies^18^ among women who have overweight or obesity. While diet and lifestyle interventions were associated with improvements in diet and modest weight loss, pregnancy, infant and childhood outcomes were not reported,^18^ or studies were inadequately powered.^18^

Our aim is to conduct a randomised controlled trial (RCT) of a comprehensive lifestyle intervention prior to conception, for women with overweight or obesity who are planning pregnancy within 2 years. We will evaluate the impact of this intervention prior to conception on maternal health and well-being prior to pregnancy; and pregnancy, birth and newborn health outcomes in a subsequent pregnancy.

## Methods and analysis

**Figure 1:**
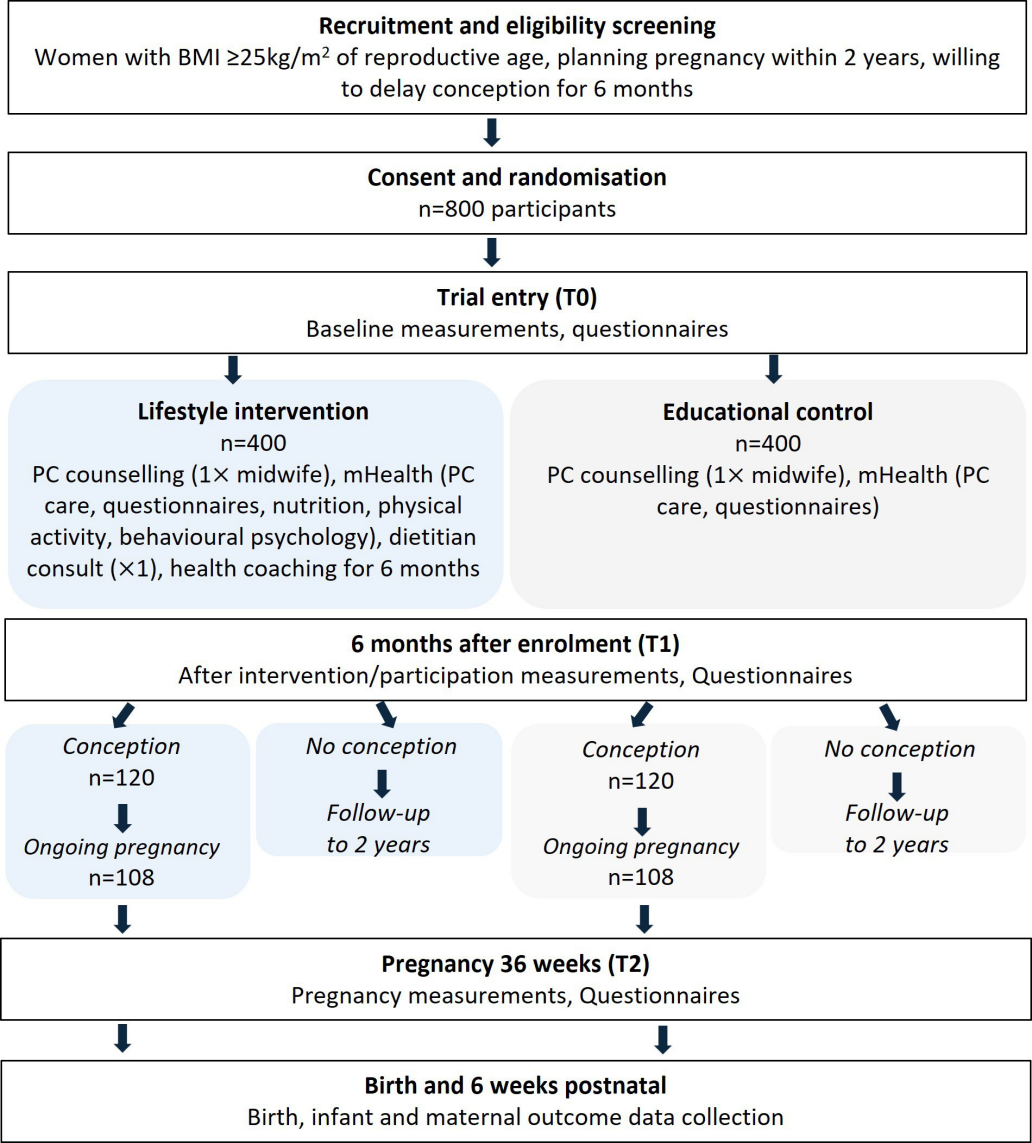
Participant flow chart for the Begin Better randomised controlled trial. PC, pre-conception.

This protocol has been reported following the Standard Protocol Items: Recommendations for Interventional Trials guidelines.^19 20^ Figure 1 shows the participant flow chart for the Begin Better RCT. The study will address prevention of adverse maternal and child health associated with overweight and obesity.

### Study design

Prospective RCT.

### Study setting

The interventional RCT will be conducted in South Australia, Australia.

### Patient and public involvement

Participants were involved in the design and conduct of the research. We conducted interviews with women to examine their perspectives of barriers, enablers and strategies for addressing overweight and obesity before conception.^21^ We then used an Information-Motivation-Behavioural Skills model to inform further interviews to determine women’s emotional and social contexts, knowledge, motivations, skills and self-efficacy in making healthy change.^22^ This work guided the planning and design process to develop an evidence-informed mHealth intervention^23^ for this RCT.

### Inclusion criteria

Women with BMI ≥25 kg/m^2^, who are of reproductive age and are planning pregnancy within 2 years following recruitment are eligible to participate.

### Exclusion criteria

Women with BMI <25 kg/m^2^, aged <18 years, who are currently pregnant or have an infant younger than 8 weeks of age, or who are unable to provide written informed consent are ineligible.

### Consent and randomisation

Women responding to social media recruitment advertising will be given trial information and counselled before providing informed written consent, including provisions for contact and additional consent to participate in any ancillary studies. Eligibility will be confirmed by research staff at a trial entry appointment (T0). Women will be randomised, with group assignment in a 1:1 ratio, using an on-line randomisation service based in the Department of Obstetrics and Gynaecology, The University of Adelaide, for sequence generation and ensuring allocation concealment. Demographic characteristics and previous birth outcomes will be collected at this appointment.

The randomisation schedule will use balanced variable blocks of size 2 and 4 allocations, prepared by an investigator not involved with recruitment or clinical care, with stratification for parity (0 vs 1+) and BMI (25.0–29.9 kg/m^2^ vs≥30 kg/m^2^). Eligible women will be randomised to the ‘Educational Control Group’ or to the ‘Pre-pregnancy Lifestyle Intervention Group’. Blinding of participants is not possible given the nature of the intervention, but outcome assessors and data analysts will be blinded to treatment allocation.

The current protocol is V.2.5, dated 12 May 2021. The first participant was enrolled in June 2021 and recruitment is anticipated to be completed in December 2025.

### Treatment schedules

Women randomised to the ‘Educational Control Group’ will attend (in person or virtual) a pre-conception health consultation with a research midwife, consistent with clinical guidelines.^24^ Advice relating to health behaviours will include cessation of smoking and illicit drugs, alcohol intake, folate supplementation, immunisations and cervical cytology. Limited information will be provided about obesity in pregnancy, nutrition, exercise and weight management, consistent with current guidelines. Women will have restricted access to the mHealth application,^23^ to receive the pre-conception care content, to maintain contact with research staff and to complete study questionnaires.

Women randomised to the ‘Pre-pregnancy Lifestyle Intervention Group’ will attend (in person or virtual) a pre-conception health consultation with a research midwife, consistent with clinical guidelines, as outlined above. Additionally, women will have an initial consultation with a research dietitian, during which a detailed dietary intake and lifestyle history will be obtained and individualised nutrition-related health goals discussed. Engagement with trained study health coaches will be in-person, via telephone, Zoom or Facetime, across the 6-month intervention period, with a focus on problem solving, revision of goals, and specifically to reflect on behavioural strategies that were effective, and those that posed a barrier to change. Women will also have unrestricted access to the specifically designed mHealth application as described above. This will include additional modules on nutrition, physical activity and behavioural psychology, as well as interactive goal-setting tasks, delivered bi-weekly throughout the 6-month intervention period.^23^

#### Ongoing follow-up of all women in both treatment groups

After the 6-month intervention period, all women will be followed for a maximum of 3.5 years to engage with those who conceive, and record pregnancy and birth outcomes to 6 weeks postpartum. Women will be contacted by phone every 2–3 months to promote participant retention.

All women will be asked to complete the Harvard Semi-quantitative Food Frequency questionnaire,^25^ the Short Questionnaire to Assess Health-enhancing Physical Activity (SQUASH)^26^ and quality of life assessments (Short Form-12 Health Survey (SF-12)^27^ and Depression Anxiety Stress Scales (DASS21)),^28^ in addition to having their weight recorded at trial entry (T0), at the completion of the 6-month intervention (T1) and at 36 weeks gestation (T2). After birth, information will be obtained relating to pregnancy, birth and infant outcomes from participant interview and medical records.

#### Study endpoints

The primary outcome is birth weight z-score. Birth weight will be converted to a z-score using population birth weight means and SD for infant sex and gestational age.^29^ While providing a robust indicator of future child obesity risk,^30^ it also controls for any confounding which may arise due to any effect of the intervention on gestational age at birth.

A range of secondary study outcomes will be collected, including:

1. Maternal pre-conception health: systolic and diastolic blood pressure, blood pressure change from trial entry to the completion of the 6-month intervention/participation period, time to conceive, need for assisted reproduction and weight change before conception.
2. Maternal pregnancy and birth outcomes: miscarriage, GWG,^31^ hypertension and pre-eclampsia,^32^ GDM,^33^ skinfold measurements at 36 weeks gestation, induction of labour, caesarean section, postpartum haemorrhage, perineal trauma, infection, endometritis, breastfeeding and maternal death. All pregnant women in South Australia are offered a fasting oral glucose tolerance test at 28 weeks of pregnancy to identify women who have GDM.
3. Maternal diet and physical activity: measured by the Harvard Semi-quantitative Food Frequency Questionnaire^25^ and SQUASH^26^ at trial entry (prior to conception), 6 months after trial entry (completion of the intervention/participation period) and at 36 weeks of pregnancy for women who become pregnant.
4. Maternal quality of life and emotional well-being: assessed using SF-12^27^ and DASS21.^28^ Measured at trial entry (prior to conception), 6 months after trial entry at the completion of the intervention/participation period and at 36 weeks of pregnancy for women who become pregnant.
5. Neonatal outcomes: obtained from maternal report and medical records, including preterm birth before 37 weeks, perinatal death, birth weight >4.0 kg, birth weight <2.5 kg, small and large for gestational age (<10th centile and >90th centile respectively, for age and sex), nursery admission, hypoglycaemia, hyperbilirubinaemia, birth trauma, shoulder dystocia, respiratory distress, infection and other neonatal complications.
6. Infant growth and development at age 6 months and 18 months as measured by questionnaires completed by the woman relating to infant feeding practices (including breast and formula feeding, and introduction of solids), infant development (as measured by the Ages and Stages Questionnaire),^34^ blood pressure, and infant height, weight and anthropometric assessment (including skin-fold thickness, body circumferences, and bioimpedance assessment of fat mass and adiposity).^35 36^
7. Biomarkers of maternal adipoinsular axis function and cardiometabolic risk. We will collect a blood sample from women at trial entry, at the end of the intervention or 6 months after trial entry for the control group, and for women in the study who conceive at 36 weeks of pregnancy. We will assess adipoinsular axis function and cardiometabolic risk, including fasting glucose, insulin, total cholesterol, high-density lipoprotein, triglycerides, non-esterified fatty acids, C-reactive protein and inflammatory cytokines.^37^
8. Maternal risk of depression will be assessed postnatally using the Edinburgh Postnatal Depression Scale^38^ when the child is aged 6 months and 18 months.

The primary paper from this RCT will report on the primary outcome (birth weight z-score) and other pregnancy and birth-related outcomes. Secondary outcomes, including longitudinal child follow-up, maternal postnatal mental health and assessment of maternal biomarkers for adipoinsular axis function and cardiometabolic risk, will be reported in subsequent publications.

### Data and safety management

Participant study data will be entered manually by research assistants or automatically transferred directly from electronic survey software (REDCap, Vanderbilt University, Tennessee) into a password-protected electronic database (Microsoft Access, Microsoft Corporation). Data will be stored on a password-protected secure server located in Australia and only researchers from the study will have access to the data. A data management plan was created prospectively and is stored via the internal research data planner tool on The University of Adelaide website.

The sharing of de-identified individual clinical trial participant-level data is intended. Before any data are shared, proposals will be reviewed by the study investigators and a data sharing agreement made with the research institution. Data will be shared on a secure registry, and as the lead investigator, JMD will act as guarantor to the final dataset. The statistician will be responsible for the development of the statistical analysis plan, prior to the conduct of any statistical analyses.

A data and safety monitoring board (DSMB) will monitor efficacy (or futility) and adverse effects. Based on these considerations, the DSMB may recommend that the protocol be modified or that the Begin Better randomised trial be terminated. The DSMB will consist of experts in relevant obstetrics, neonatology and research methodology. All adverse events involving women and infants enrolled in the trial will be reviewed by a multidisciplinary committee, who will be blinded to treatment allocation, in order to clarify cause. These data will be made available for the DSMB.

### Sample size estimation

The primary outcome is birth weight z-score. The original calculation used data available from a similar cohort of pregnant women with overweight and obesity^5^ and estimated the mean birth weight z-score to be 0.43 (SD 1.09). To detect a clinically meaningful difference in birth weight z-score of 0.3 SD, we estimated randomisation of 864 women to obtain pregnancy outcome data from 418 women (80% power; two-sided alpha=0.05). This included an anticipated pregnancy rate of approximately 55%, an early pregnancy loss rate of approximately 12%, with a 10% loss to follow-up and 2% of births from a multiple pregnancy. The sample of 864 women at intervention completion had 99% power to detect a pre-pregnancy difference in weight of 3 kg (two-sided alpha=0.05).^39^

We have re-estimated the sample size after noting that the pregnancy rate and losses to follow-up were lower than originally anticipated, and to account for funding constraints. To detect a clinically meaningful difference in birth weight z-score of 0.4 SD, we will require birth weight outcomes for 236 babies (80% power; two-sided alpha=0.05). To achieve this, we will randomise 800 women (anticipated pregnancy rate of approximately 33%, and early pregnancy loss rate of approximately 10%). The sample of 800 women at intervention completion prior to conception has 99% power to detect a pre-pregnancy difference in weight of 3 kg (two-sided alpha=0.05).

### Analysis and reporting of results

We will examine baseline characteristics of all randomised women, as an indication of comparable treatment groups. Primary and secondary outcomes will be analysed on an intention-to-treat basis, according to treatment allocation at randomisation, with adjustment for stratification variables of BMI and parity, and pre-specified prognostic baseline variables. Statistical significance will be indicated by p<0.05 (two-sided). Generalised estimating equations or random effects will be used to account for non-independence between outcomes of infants from multiple births.

Missing data will be handled through multiple imputation using the fully conditional specification (chained equations) method to create 100 complete datasets for analysis of primary and secondary outcomes, with sensitivity analyses using available data only, different imputation models or assuming that data are Missing Not at Random.

Analyses will be conducted according to a detailed statistical analysis plan, which will be finalised and approved by investigators before any analysis is performed. The estimands framework^40^ will be used to specify the intervention, target population, outcome definition and population-level summary for the primary and each secondary outcomes, along with any relevant intercurrent events (post-randomisation events which affect the value or existence of the outcome), assumptions made by the chosen analysis approach and planned sensitivity analyses. In general, the analysis population will be that best approximating the intention-to-treat population for each outcome.

For outcomes that are only relevant to the subgroup of women who achieve an established pregnancy, sensitivity analyses will explore the effect of treatment among women who would have achieved pregnancy under either treatment group assignment.^41^

Binary outcomes will be assessed using log binomial regression models, and with results as relative risks and 95% CI. Time to conception will be assessed using Kaplan-Meier estimates and/or Cox proportional hazards models. Continuous outcomes will be assessed using linear regression models, with results reported as differences in means and 95% CI. Longitudinal outcomes will be assessed using mixed effects models and include a time-by-treatment interaction term.

## Ethics and dissemination

Ethics approval for this randomised trial has been obtained from the Women’s and Children’s Hospital Ethics Committee (HREC/17/WCHN/177; approved 17 August 2018; HealthWCHNResearch@sa.gov.au). The trial is registered with the Australian and New Zealand Clinical Trials Registry (ACTRN 12621000128897; date registered: 8 February 2021). The research team will share important protocol modifications with relevant parties, including investigators, trial registries and trial participants as appropriate and in compliance with relevant laws and regulations. Findings will be disseminated widely via journal publication and conference presentation(s), with authorship determined using the International Committee of Medical Journal Editors recommendations. The primary paper will report on the primary outcome (birth weight z-score) and other pregnancy and birth-related outcomes, with other longitudinal secondary outcomes reported in subsequent publications. Participants will be informed of the results.

## Data Availability

No data are available.
