# Supplementary material for: Pre-conception lifestyle intervention to optimise maternal health for a better start to life: the BEGIN BETTER prospective randomised controlled trial protocol
Source: BMJ Open. 2025 Oct 15;15(10):e105478. doi: 10.1136/bmjopen-2025-105478 (PMC12530384; doi:10.1136/bmjopen-2025-105478)
Supplement: Supplementary file 1 [file bmjopen-2025-105478.reviewer_comments.pdf]

PEER REVIEW HISTORY

BMJ Open publishes all reviews undertaken for accepted manuscripts. Reviewers are asked to complete a checklist review form and are provided with free text boxes to elaborate on their assessment. These free text comments are reproduced below.

ARTICLE DETAILS

Title (Provisional)

Pre-conception lifestyle intervention to optimise maternal health for a better start to life: the BEGIN BETTER prospective randomised controlled trial protocol

Authors

Mitchell, Megan; Deussen, Andrea R; Louise, Jennie; Poprzeczny, Amanda J; Keir, Amy K; Briley, Annette L; Oxlad, Melissa; Dodd, Jodie M

VERSION 1 - REVIEW

**Reviewer**	**1**
**Name**	**Winichagoon, Pattanee**
**Affiliation**	**Mahidol University**
**Date**	**14-Aug-2025**
**COI**	**None**

This study protocol is very well thought out, and the described in the MS. However, there is no statistical analysis plan included. In addition, cost analysis is mentioned in the abstract, but nothing is described in the MS. Also, a suggestion on qualitative study of the lessons from intervention which could be useful for scaling up, if it is successful. Specific comments are as follows.

1. Intervention

a. Since it has an individualized and interactive component, it would be very useful to document qualitatively in a small subsample, of the experiences/lessons learned in terms of challenges, facilitating or responsiveness to the designed intervention.

2. Outcomes:

3. Primary outcome: For weight-z-score, which reference is used for this calculation, local (Australian) or international reference e.g., Intergrowth 21.

4. Secondary outcomes:

a. Maternal pregnancy – for hypertension/pre-eclampsia, is it monitored regularly as per study visit protocol?

b. Costs of healthcare (mentioned in the Abstract): Please clarify/give more explanation on what will be included, i.e., costs to the health services; costs to the mothers/clients. In addition, will it be just costs in monetary terms, or will be a sort of cost-effectiveness in which case, the methods to be used should be elaborated, e.g., cost for which outcome(s) averted due to the intervention.

**Reviewer**	**2**
**Name**	**Yan, Weili**
**Affiliation**	**Children's Hospital of Fudan University**
**Date**	**03-Sep-2025**
**COI**	**None**

This is an important randomized trial evaluating whether comprehensive pre-conception lifestyle interventions aimed at maternal weight loss can reduce birth weight. The study is large-scale, with a prolonged intervention and follow-up period. Such a design inevitably encounters several challenges: ensuring intervention fidelity and participant adherence, minimizing contamination, accommodating unexpected pregnancies during the study, mitigating loss to follow-up, and accounting for the inherent uncertainty in the intervention effect size.

For consideration:

1. Eligibility criteria

Restricting participants to ≥18 years and BMI ≥25 are inclusion criteria. Below age of 18 and BMI of 25 should be removed.

2. Randomization

Clarify the randomization procedure. If block randomization is used, state the block size(s) and whether block sizes will be variable. If stratification is planned, specify the stratification factors and the target proportions within each stratum.

3. Primary outcome

Provide the reference standards (e.g., INTERGROWTH-21st, WHO, or local growth charts) that will be used to normalize birth-weight data.

4. Statistical analysis

Detail the primary analysis strategy to the primary outcome, birth weight Z score, beyond implying it as one of “continuous outcome”, since the result of which will be used for drawing the conclusion. Specify:

• Define the analysis set, how the ITT population will be constructed given the anticipated low pregnancy rate—whether imputation will be used. These decisions must align with the assumptions used in the sample-size calculation.

•Major sensitivity analyses under consideration.

A comprehensive statistical analysis plan will undoubtedly follow, but clarifying the above points in the protocol will strengthen both scientific rigor and feasibility.

5. Contamination control

Describe concrete measures to prevent participants in the control arm from receiving or adopting the active intervention components.

6. Generalizability

Discuss how the intervention package could be translated to routine pre-conception care.

VERSION 1 - AUTHOR RESPONSE

Reviewer: 1

Dr. Pattanee Winichagoon, Mahidol University

Comments to the Author:

1. **This study protocol is very well thought out, and the described in the MS. However, there is no statistical analysis plan included. In addition, cost analysis is mentioned in the abstract, but nothing is described in the MS. Also, a suggestion on qualitative study of the lessons from intervention which could be useful for scaling up, if it is successful. Specific comments are as follows.**

Thank you for these comments:
The statistical analysis plan has been referred to in the manuscript (under 'Data and safety management') and will be completed, dated and signed consistent with best practice and the 2025 revised CONSORT Statement (https://www.consort-spirit.org/item3-accesstotrialprotocol), prior to the finalisation of the dataset for analyses. The Statistical Analysis Plan will be made available for access, either as supplementary material for the main study paper, or in an open online repository. Additional exploratory and descriptive side studies are underway including a qualitative analysis of the experience of participants in the trial and will be reported separately to the primary trial. Costs of healthcare may be considered as a side study following publication of the primary and secondary outcomes. It was included in the abstract in error and has now been removed.

Intervention

a. **Since it has an individualized and interactive component, it would be very useful to document qualitatively in a small subsample, of the experiences/lessons learned in terms of challenges, facilitating or responsiveness to the designed intervention.**

Additional exploratory and descriptive side studies are underway including a qualitative analysis of the experience of participants in the trial and will be reported separately to the primary trial.

Outcomes:

a. **Primary outcome:**
   - **For weight-z-score, which reference is used for this calculation, local (Australian) or international reference e.g., Intergrowth 21.**

We will use Intergrowth 21[#] to calculate birth weight z-scores. This is now referenced for the description of the primary outcome.

Papageorghiou et al; International Fetal and Newborn Growth Consortium for the 21(st) Century (INTERGROWTH-21(st)). The INTERGROWTH-21st fetal growth standards: toward the global integration of pregnancy and pediatric care. Am J Obstet Gynecol. 2018 Feb;218(2S):S630-S640.

b. **Secondary outcomes:**
   - **Maternal pregnancy – for hypertension/pre-eclampsia, is it monitored regularly as per study visit protocol?**

Routine antenatal care in South Australia includes measurement of blood pressure at all antenatal visits, hospital stays including birth and postpartum until hospital discharge. Study outcome data related to hypertension in pregnancy and pre-eclampsia is obtained from the woman's clinical record.

c. **Costs of healthcare (mentioned in the Abstract): Please clarify/give more explanation on what will be included, i.e., costs to the health services; costs to the mothers/clients. In addition, will it be just costs in monetary terms, or will be a sort of cost-effectiveness in which case, the methods to be used should be elaborated, e.g., cost for which outcome(s) averted due to the intervention.**

Costs of healthcare may be considered as a side study following publication of the primary and secondary outcomes. It was included in the abstract in error and has now been removed.

<u>Reviewer: 2</u>

Dr. Weili Yan, Children's Hospital of Fudan University

Comments to the Author:
This is an important randomized trial evaluating whether comprehensive pre-conception lifestyle interventions aimed at maternal weight loss can reduce birth weight. The study is large-scale, with a prolonged intervention and follow-up period. Such a design inevitably encounters several challenges: ensuring intervention fidelity and participant adherence, minimizing contamination, accommodating unexpected pregnancies during the study, mitigating loss to follow-up, and accounting for the inherent uncertainty in the intervention effect size.

For consideration:

1. Eligibility criteria
Restricting participants to ≥18 years and BMI ≥25 are inclusion criteria. Below age of 18 and BMI of 25 should be removed.

Thank you for this suggestion, whilst we agree specifying these as exclusions is somewhat redundant, for absolute clarity we prefer to retain.

2. Randomization
Clarify the randomization procedure. If block randomization is used, state the block size(s) and whether block sizes will be variable. If stratification is planned, specify the stratification factors and the target proportions within each stratum.

We have updated the following sentence regarding block randomisation:
"The randomisation schedule will use balanced variable blocks of size 2 and 4"

The manuscript details the following stratification:
'…..prepared by an investigator not involved with recruitment or clinical care, with stratification for parity (0 vs 1+), and BMI (25.0-29.9kg/m^2 vs ≥30kg/m^2).'

No target proportions within strata are specified as stratification is solely to ensure that treatment groups are balanced in relation to characteristics known to be strongly related to outcome, so that the difference between groups is not influenced by chance imbalances. The randomisation schedule contained many more randomisations than expected within each stratum to allow for some strata to be larger than others due to their distribution in the population.

3. Primary outcome
Provide the reference standards (e.g., INTERGROWTH-21st, WHO, or local growth charts) that will be used to normalize birth-weight data.

We will use Intergrowth 21[#] to calculate birth weight z-scores. This is now referenced for the description of the primary outcome.
Papageorghiou et al; International Fetal and Newborn Growth Consortium for the 21(st) Century (INTERGROWTH-21(st)). The INTERGROWTH-21st fetal growth standards: toward the global integration of pregnancy and pediatric care. Am J Obstet Gynecol. 2018 Feb;218(2S):S630-S640.
4. Statistical analysis

a. **Detail the primary analysis strategy to the primary outcome, birth weight Z score, beyond implying it as one of "continuous outcome", since the result of which will be used for drawing the conclusion. Specify:**

The following detail has been added to the manuscript, with the appropriate reference:
"Analyses will be conducted according to a detailed Statistical Analysis Plan, which will be finalised and approved by investigators before any analysis is performed. The estimands framework will be used to specify, for the primary and each secondary outcomes, the intervention, target population, outcome definition, and population-level summary, along with any relevant intercurrent events (post-randomisation events which affect the value or existence of the outcome), assumptions made by the chosen analysis approach, and planned sensitivity analyses. In general, the analysis population will be that best approximating the Intention-to-Treat population for each outcome."*

b. **Define the analysis set, how the ITT population will be constructed given the anticipated low pregnancy rate—whether imputation will be used. These decisions must align with the assumptions used in the sample-size calculation.**

It is planned that the Statistical Analysis Plan will specify:
For the primary outcome, the target population is infants of women who would have conceived and delivered an infant (> 20 weeks' gestational age) regardless of group allocation. The analysis population will consist of all infants delivered after 20 weeks' GA, analysed in the groups to which their mothers were randomised regardless of actual receipt of intervention. Multiple imputation will be used to impute missing outcome data for participants who had pregnancies of at least 20 weeks' gestation. Outcomes will not be imputed for participants who did not become pregnant, or who suffered pregnancy loss prior to 20 weeks' gestation, as these outcomes are undefined rather than missing.
Because failure to conceive, and pregnancy loss prior to 20 weeks, occur after randomisation, and are potentially affected by the intervention, it is possible that the analysis population does not correspond exactly to the target population: specifically, some participants will have an infant who would not have conceived (or maintained a pregnancy) if they had been allocated to the other group. The analysis approach therefore assumes that the mean birthweight z-score will not be affected by this difference.

c. **Major sensitivity analyses under consideration.**

It is planed that the Statistical Analysis Plan will specify:
In order to assess this assumption, a sensitivity analysis will be performed using the Principal Stratum strategy, in which the effect of the intervention is estimated in the principal stratum consisting of (infants of) participants who would have conceived, and would not have suffered pregnancy loss prior to 20 weeks, in either intervention group. Additional sensitivity analyses will be performed to assess the potential impact of other factors (for example, missing information on pregnancy or delivery), and outcome data missing not at random.

d. **A comprehensive statistical analysis plan will undoubtedly follow, but clarifying the above points in the protocol will strengthen both scientific rigor and feasibility.**

The statistical analysis plan has been referred to in the manuscript (under 'Data and safety management') and will be completed, dated and signed consistent with best practice and the 2025 revised CONSORT Statement (https://www.consort-spirit.org/item3-accesstotrialprotocol), prior to the finalisation of the dataset for analyses. This will allow for any emerging statistical methods to be incorporated.
The Statistical Analysis Plan will be made available for access, either as supplementary material for the main study paper, or in an open online repository.

5. Contamination control

Describe concrete measures to prevent participants in the control arm from receiving or adopting the active intervention components.

As this is a pragmatic trial in a real world setting, we are satisfied that the trial findings will reflect what may happen if such a program as Begin Better were implemented into clinical practice at a population level which may include women adopting lifestyle habits that may improve their health.

6. Generalizability

Discuss how the intervention package could be translated to routine pre-conception care.

Unfortunately in Australia, although pre-conception care is recommended, it is not well attended and usually women access this when planning for a baby in the very near future.
Realistically, we would hope that if the findings of this trial show the intervention to be beneficial it would encourage further research in this population to strengthen the evidence base for such interventions to be made available to the wider population of women of reproductive age.

Reviewer: 1
If you have selected 'Yes' above, please provide details of any competing interests.: Not applicable

Reviewer: 2
If you have selected 'Yes' above, please provide details of any competing interests.: Not applicable

VERSION 2 - REVIEW

**Reviewer**	**2**
**Name**	**Yan, Weili**
**Affiliation**	**Children's Hospital of Fudan University**
**Date**	**23-Sep-2025**
**COI**	

All of my comments have been addressed.
